# Intracellular Ca^2+^ Stores and Ca^2+^ Influx Are Both Required for BDNF to Rapidly Increase Quantal Vesicular Transmitter Release

**DOI:** 10.1155/2012/203536

**Published:** 2012-07-03

**Authors:** Michelle D. Amaral, Lucas Pozzo-Miller

**Affiliations:** Department of Neurobiology, Civitan International Research Center, The University of Alabama at Birmingham, SHEL-1002, 1825 University Boulevard, Birmingham, AL 35294-2182, USA

## Abstract

Brain-derived neurotrophic factor (BDNF) is well known as a survival factor during brain development as well as a regulator of adult synaptic plasticity. One potential mechanism to initiate BDNF actions is through its modulation of quantal presynaptic transmitter release. In response to local BDNF application to CA1 pyramidal neurons, the frequency of miniature excitatory postsynaptic currents (mEPSC) increased significantly within 30 seconds; mEPSC amplitude and kinetics were unchanged. This effect was mediated via TrkB receptor activation and required both full intracellular Ca^2+^ stores as well as extracellular Ca^2+^. Consistent with a role of Ca^2+^-permeable plasma membrane channels of the TRPC family, the inhibitor SKF96365 prevented the BDNF-induced increase in mEPSC frequency. Furthermore, labeling presynaptic terminals with amphipathic styryl dyes and then monitoring their post-BDNF destaining in slice cultures by multiphoton excitation microscopy revealed that the increase in frequency of mEPSCs reflects vesicular fusion events. Indeed, BDNF application to CA3-CA1 synapses in TTX rapidly enhanced FM1-43 or FM2-10 destaining with a time course that paralleled the phase of increased mEPSC frequency. We conclude that BDNF increases mEPSC frequency by boosting vesicular fusion through a presynaptic, Ca^2+^-dependent mechanism involving TrkB receptors, Ca^2+^ stores, and TRPC channels.

## 1. Introduction

Brain-derived neurotrophic factor (BDNF) is a member of the neurotrophic factor family and is well known as a survival factor and chemoattractant during development of the central nervous system [1]. In recent years, however, it has been shown that BDNF also plays a significant role modulating synaptic plasticity in the hippocampus [2–6]. These functions include the enhancement of synaptic transmission at excitatory synapses [7] and alterations of dendritic architecture [8], for example, increasing dendritic spine density [9–12]. BDNF exerts its effects both at the presynapse and the postsynapse: at the presynaptic level, BDNF modulates quantal synaptic transmission, and at hippocampal CA3-CA1 pyramidal neuron synapses, this is manifested as transient increases in the frequency of miniature synaptic currents.

Acute application of BDNF enhances spontaneous and evoked glutamatergic excitatory postsynaptic currents in cultured neurons [13–17]. Long-term treatment of postnatal hippocampal slice cultures with BDNF increases the frequency of miniature excitatory postsynaptic currents (mEPSCs) recorded from CA1 pyramidal neurons, without affecting their amplitude or kinetics [12]. The latter effect of BDNF was specific on a rapidly recyclable pool of vesicles. Indeed, BDNF selectively increased the density of synaptic vesicles docked at the active zone of asymmetric spine synapses, without affecting those in the main vesicle cluster [12]. Furthermore, BDNF selectively enhanced evoked and spontaneous FM1-43 destaining in acute hippocampal slices, but only when presynaptic terminals were dye-loaded with a hyperosmotic shock using sucrose [18], a manipulation that only engages the readily releasable pool of vesicles [19, 20].

Two potential mechanisms mediating the BDNF-induced increase in transmitter release include synapsin-I phosphorylation [21] and TrkB-initiated PLC*γ* activation leading to Ca^2+^ mobilization from IP3-sensitive stores [22]. Previous studies have suggested that Ca^2+^ influx following Ca^2+^ store depletion may contribute to spontaneous vesicular release [23]. However, studies regarding BDNF-mediated increases in mEPSC frequency in dissociated cultured neurons have concluded that this effect is dependent upon Ca^2+^ release from intracellular stores only [16]. We herein report that BDNF activation of TrkB receptors rapidly enhances spontaneous quantal transmitter release in CA1 pyramidal neurons of hippocampal slice cultures by Ca^2+^ mobilization from intracellular stores, a signal further amplified by Ca^2+^ entry through TRPC plasma membrane channels. Additionally, BDNF causes immediate FM dye destaining in the absence of action potentials, directly proving its presynaptic effect and the vesicular origin of postsynaptic quantal responses. The rate of BDNF-induced destaining was similar for two FM dyes with different membrane departition rates, suggesting that BDNF increases mEPSC frequency by either full-fusion events or through fusion pores with limited FM dye permeability.

## 2. Materials and Methods

### 2.1. Organotypic Slice Culture

All animal procedures strictly adhered to national and international guidelines for the ethical use of research animals. The Institutional Animal Care and Use Committee (IACUC) of The University of Alabama at Birmingham (UAB) reviews and approves all animal procedures described in the present paper on an annual basis. Briefly, hippocampi were dissected from anesthetized postnatal days 7–11 Sprague-Dawley rats (Harlan, Indianapolis, IN, or Charles River Laboratories, Wilmington, MA, USA) and cut transversely into 400 *μ*m thick slices using a custom-made wire slicer fitted with 20 *μ*m thick gold-coated platinum wire [24]. Hippocampal slices were individually plated on Millicell-CM filter inserts (Millipore, Billerica, MA, USA) and cultured in 36°C, 5% CO_2_, 98% relative humidity incubators (Thermo-Forma, Waltham, MA, USA). Slices were maintained in culture media (Neurobasal-A plus B27; Invitrogen, Carlsbad, CA), containing 20% equine serum for the first 4 days in vitro (div). To avoid the confounding effects of hormones and growth factors in the serum, its concentration was gradually reduced over a period of 48 h starting at 4 div (24 h each in 10 and then 5% serum). After a period of 24 h in serum-free media (Neurobasal-A plus B27), 7–10 div slices were used for electrophysiology [12].

### 2.2. Whole-Cell Intracellular Recordings

Individual 7–10 div slices were transferred to a recording chamber mounted on a fixed-stage upright microscope (Axioskop FS; Zeiss, Oberkochen, Germany) and continuously perfused (2mL/min) with artificial CSF (aCSF) at room temperature (24°C) containing the following (in mM): 124 NaCl, 2 KCl, 1.24 KH_2_PO_4_, 1.3 MgSO_4_, 17.6 NaHCO_3_, 2.5 CaCl_2_, 10 glucose, and 29.2 sucrose (310–320 mOsm). aCSF was bubbled with 95% O_2_/5% CO_2_, pH 7.4. Superficial CA1 pyramidal neurons were visualized with a water-immersion 63x objective (0.9 numerical  aperture (NA)) using infrared differential interference contrast (IR-DIC) microscopy. Whole-cell intracellular recordings were performed as previously described [10, 24].

Human recombinant mature BDNF (supplied by Amgen, Thousand Oaks, CA, or purchased from Promega, Madison, WI, USA) was pressure applied to hippocampal slices from glass pipettes (~5 MΩ) using a Picospritzer III (Parker Hannifin, Cleveland, OH, USA) as described [10, 22].

### 2.3. Loading FM Dyes into Presynaptic Terminals in Slice Cultures

Hippocampal slices, maintained in serum-free medium for 10 to 12 days, were labeled with either FM1-43 or FM2-10 dyes. The total recycling pool of vesicles was loaded via endocytosis by incubating slices in 40 mM K^+^ aCSF containing 0.5 *μ*M TTX and either 15 *μ*M FM1-43 or 100 *μ*M FM2-10; the solution had been prewarmed to 37°C and was under constant aeration (95% O_2_/5% CO_2_) throughout the 15 min incubation. Individual slices were then placed in standard aCSF containing 0.5 *μ*M TTX and either 5 *μ*M FM1-43 or 100 *μ*M FM2-10 for 3 minutes, then in dye-free, nominally zero Ca^2+^ aCSF containing 0.5 *μ*M TTX for a 30-minute washout. Imaging was conducted in standard aCSF containing 0.5 *μ*M TTX, and FM dye fluorescence intensity was monitored during pressure application of BDNF from glass pipettes (~5 MΩ) using a Picospritzer-III (Parker Hannifin, Cleveland, OH, USA).

### 2.4. Multiphoton Excitation Microscopy of FM-Labeled Terminals in Slice Cultures

 FM dye fluorescence images were collected with a 60x, 0.9 NA water-immersion objective (LUMPlanFI/IR2; Olympus, Melville, NY, USA), using a custom-built multiphoton excitation laser scanning microscope consisting of an upright BX50WI microscope and a modified FV300 scanhead (Olympus). A Ti-sapphire laser pumped by a 12 W diode Ar laser (Chameleon; Coherent, Santa Clara, CA, USA) was tuned to 840 nm center wavelength. Infrared laser intensity was controlled by an external Pockels cell (Conoptics, Danbury, CT, USA) before entering the scanhead. Infrared-filtered FM dye fluorescence emission was detected by a GaASP photomultiplier (PMT) (H7422P-40; Hamamatsu) in nondescanned mode. The lowest intensity necessary for adequate signal-to-noise ratio was used to avoid photodamage and FM dye bleaching. Average power in the back focal plane of the objective never exceeded 50 mW. IR-filtered FM dye fluorescence was detected by laser-scanning microscopy and time-lapse image acquisitions were controlled by FluoView-Tiempo software (Olympus).

Full-frame images (512 × 512 pixels, ~72 × 72 *μ*m) were acquired every 1.1 second. All image fields were obtained from within ~200 *μ*m of the slice surface, typically ~25–150 *μ*m deep. Ten image frames were acquired before stimulation and used to determine baseline fluorescence (*F*_*b*_). Once stimulation was delivered, FM dye emission intensity was measured, and changes were expressed as Δ*F*/*F*_*b*_. FM dye bleaching was measured by image sequences of similar duration but without stimulation and was less than 5% over the 30-minute long movies. FM dye fluorescence intensity was measured from visually identified puncta or clusters of several puncta that could be individually tracked in a single *z*-plane (focal) section over time. The fluorescence intensity of each of these regions-of-interest (ROI) was individually measured, normalized, and plotted over time, after background and bleaching subtraction [18, 25–28]. Fluorescence changes over time in single puncta were analyzed using ImageJ [29]. All data analyses were performed blindly and included only those FM puncta, whose fluorescence intensity was more than two times the SD of the baseline FM dye fluorescence intensity [28]. Identical ROIs with a constant diameter of 1 *μ*m (seven pixels) were used to select individual puncta and ensure consistent analysis over time and regions in a slice. Data from experiments where lateral displacement (*x*-*y*) or focal drift in the *z*-axis of FM dye puncta occurred, were discarded. To avoid imaging nonselective FM staining, only puncta that showed stimulus-dependent destaining were included in the analyses.

### 2.5. Statistical Analyses

Data were analyzed using paired Student's *t*-test for comparing BDNF effects on mEPSC mean frequency in the same cells, Kolmogorov-Smirnov test for comparing median mEPSC frequencies, and unpaired Student's *t*-test for comparing FM dye destaining rates (Prism; Graph-Pad Software, San Diego, CA, USA). *P* < 0.05 was considered significant. Data are presented as mean ± SEM.

## 3. Results

Long-term (minutes to hours) exposure to BDNF induces varied effects on hippocampal neurons, ranging from modulation of synaptic transmission and plasticity to structural changes of dendrites, spines, and presynaptic terminals [3, 4, 6, 30]. In order to investigate whether short-term exposure has similar effects, we applied BDNF from picospritzer-controlled pipettes placed 100 *μ*m above hippocampal slice cultures, a configuration that prevents pressure and mechanical artifacts [10].

### 3.1. Acute Exposure to BDNF Rapidly Increases mEPSC Frequency in Hippocampal CA1 Pyramidal Neurons

**Figure 1:**
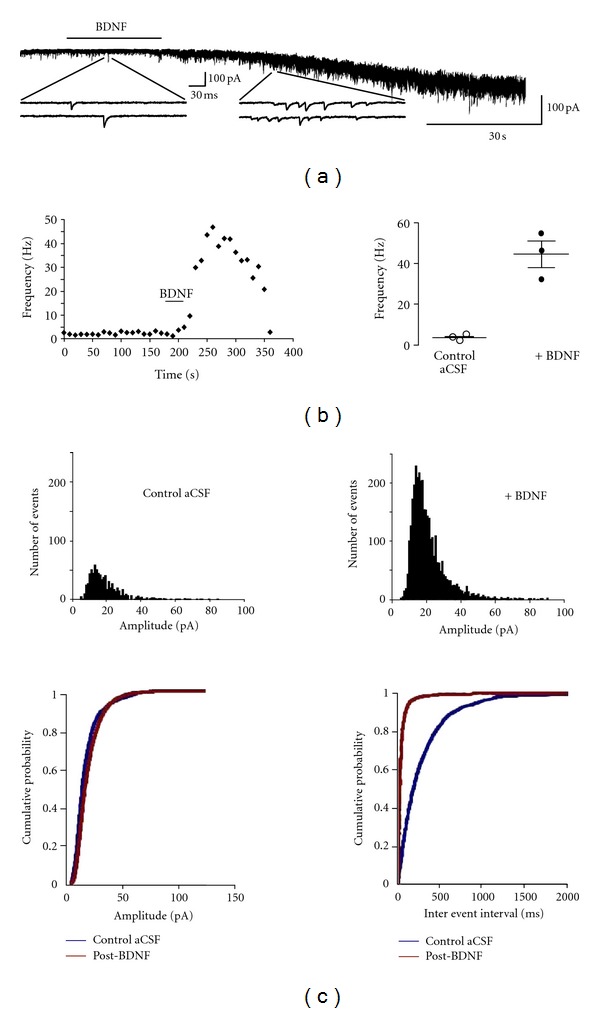
BDNF rapidly increases mEPSC frequency. (a) Representative trace demonstrating the effect of BDNF on mEPSC frequency recorded in CA1 pyramidal cells. BDNF was applied at a concentration of 100 ng/mL from a Picrospritzer-controlled pipette aimed at CA1 *striatum radiatum* in a 14 div slice culture. Insets show representative mEPSCs at higher time resolution. (b) Left: representative running average plot of mEPSC frequency as a function of time (sec); right: mean mEPSC frequency before and after BDNF application. (c) Top: tepresentative probability distributions of mEPSC amplitudes prior to and following application of BDNF; bottom: cumulative probability distributions for amplitude and interevent intervals prior to and following application of BDNF.

BDNF-containing pipettes were positioned over CA1 neuron dendrites within *stratum radiatum*, 200 *μ*m away from their cell bodies, to reproduce the spatiotemporal profile of a paracrine neuropeptide released from dense-core vesicles acting on perisynaptic receptors [31]. Under these conditions, a single BDNF puff of 30 sec duration increased the mean mEPSC frequency from 3.6 ± 0.6 Hz to 44.8 ± 6.6 Hz measured at 70 ± 10 sec following BDNF application (*n* = 3, *P* = 0.0262) (Figures 1(a) and 1(b)), which is a ~14 fold increase over the baseline levels. The BDNF-induced elevation of mEPSC frequency was evident within seconds, and lasted 176.7 ± 11.6 sec before returning to baseline levels. The median baseline mEPSC frequency was 3.9 Hz, while the peak median mEPSC frequency following BDNF application reached 21.3 Hz. A Kolmogorov-Smirnov test for differences between baseline mEPSC frequency and BDNF-induced mEPSC frequency yielded a *Z* value of 14.6 ± 0.7, which was highly significant (*P* < 0.05) (Figure 1(c)). The inward current observed in the representative example shown in Figure 1(a) is mediated by TRPC3-containing channels and was fully characterized in Amaral and Pozzo-Miller [10].

The amplitude of individual mEPSCs before and after BDNF exposure was not statistically different (20.9 ± 0.9 pA versus 22.6 ± 0.5 pA, *n* = 3, *P* > 0.05) (Figure 1(c)). Similarly, the kinetics of individual mEPSCs was not affected by BDNF (rise time: 1.7 ± 0.3 ms versus 2 ± 0.3 ms, *n* = 3, *P* > 0.05).

### 3.2. BDNF Increases mEPSC Frequency by Activation of Trk Receptors

**Figure 2:**
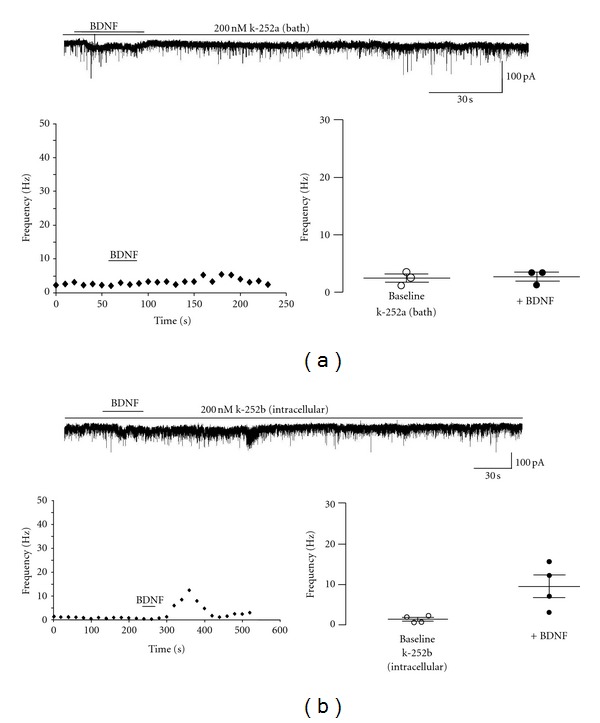
The increase in mEPSC frequency requires TrkB receptor activation. (a) Top: representative trace showing the BDNF effect in a CA1 pyramidal cell in the presence of the receptor tyrosine kinase inhibitor k-252a (200 nM); left: representative running average plot of mEPSC frequency in the presence of k252a; right: mean frequency of mEPSCs before and after BDNF in the continuous presence of k252a. (b) Top: representative trace recorded showing the BDNF effect in a CA1 pyramidal cell dialyzed with k252b (200 nM), a membrane impermeable inhibitor of the Trk receptor family; left: running average plot of mEPSC frequency in a representative k252b-loaded neuron; right: mean frequency of mEPSCs before and after BDNF in k252b-loaded neurons.

The BDNF-induced increase in mEPSC frequency was mediated by Trk receptors, as the effect did not occur after bath application of k-252a, an inhibitor of tyrosine kinases [32] (mean: 2.4 ± 0.6 Hz versus 2.6 ± 0.8 Hz, *n* = 3, *P* = 0.51; median: 2.6 Hz versus 3.3 Hz; Kolmogorov-Smirnov *Z* value: 1.9 ± 1.3) (Figure 2(a)). On the other hand, intracellular application of k-252b (an analog of lower membrane permeability) reduced but not fully prevented the BDNF effect on mEPSC frequency (mean: 1.41 ± 0.4 Hz versus 9.6 ± 2.7 Hz, *n* = 3, *P* = 0.1250; median: 1.94 Hz versus 3.59 Hz; Kolmogorov-Smirnov *Z* value: 4.31 ± 3.7), which was a 6.77 fold increase over the baseline (Figure 2(b)). The BDNF response in k-252b-loaded neurons peaked at 80 ± 40 sec following BDNF application (not different in control conditions) but lasted significantly less (76.67 ± 37.86 sec versus 176.7 ± 11.6 sec; *n* = 3, *P* = 0.0289). The persistence of such response—albeit attenuated—reflects the contribution of presynaptic Trk receptors (which are not inhibited in k-252b-loaded neurons) to the overall effect measured under control conditions. The fact that intracellular k-252b application reduced the amplitude and duration of the BDNF effect on mEPSC frequency suggests the intriguing possibility that postsynaptic Trk receptors signal retrogradely to modulate presynaptic transmitter release. Alternatively, k-252b could simply leak out of the postsynaptic cell to act on nearby presynaptic terminals. Both of these pharmacological agents were used at a concentration of 200 nM in 0.01% DMSO, which is specific for receptor tyrosine kinases of the *trk* gene family [33]. Vehicle controls revealed no effects of either extracellular or intracellular DMSO on mEPSC frequency, amplitude, and kinetics.

### 3.3. BDNF-Induced Elevation in mEPSC Frequency Is Sensitive to Manipulations of Ca^2+^ Levels

**Figure 3:**
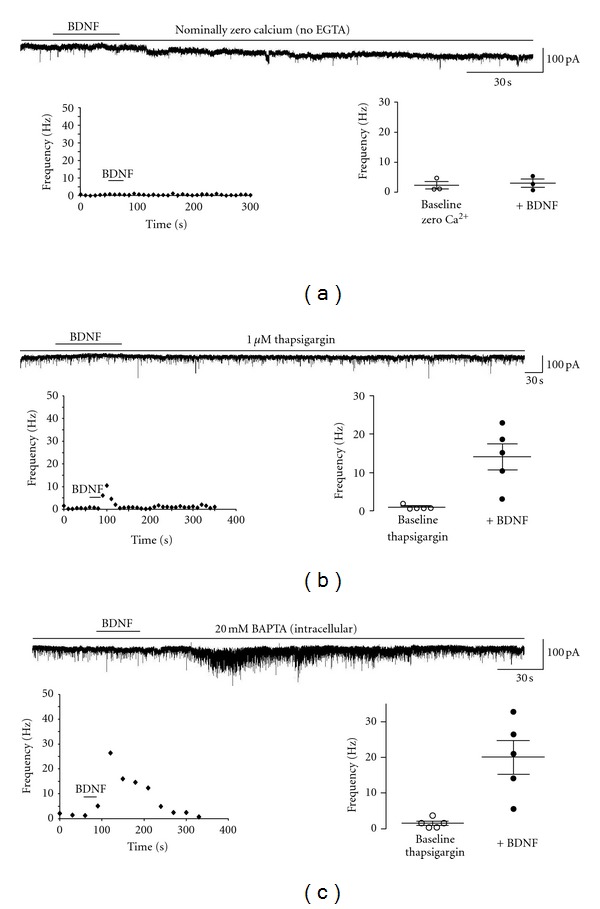
BDNF requires Ca^2+^ influx and intracellular Ca^2+^ mobilization to increase mEPSC frequency. (a) Top: representative trace from a CA1 pyramidal cell in the absence of extracellular Ca^2+^ prior to and following BDNF application; left: representative running average plot of mEPSC frequency in the absence of extracellular Ca^2+^; right: mean frequency of mEPSCs before and after BDNF in the absence of extracellular Ca^2+^. (b) Top: representative trace from a CA1 pyramidal cell in the presence of the SERCA pump inhibitor thapsigargin (1 *μ*M) prior to and following BDNF application; left: representative running average plot of mEPSC frequency in the presence of thapsigargin; right: mean frequency of mEPSCs before and after BDNF in the continuous presence of thapsigargin. (c) Top: representative trace from a CA1 pyramidal cell loaded with BAPTA prior to and following BDNF application; left: running average plot of mEPSC frequency in a representative BAPTA-loaded neuron, right: mean frequency of mEPSCs before and after BDNF in BAPTA-loaded neurons.

Since the frequency of asynchronous synaptic transmission is sensitive to changes in Ca^2+^ and BDNF signaling engages intracellular Ca^2+^ signaling, we next examined whether the BDNF effect on mEPSC frequency was sensitive to manipulations of Ca^2+^ levels. Indeed, BDNF failed to increase the mean mEPSC frequency in the absence of extracellular Ca^2+^ (2.26 ± 1.2 Hz versus 2.94 ± 1.3 Hz, *n* = 3, *P* = 0.7523) (Figure 3(a)). The median baseline frequency was 0.69 Hz, which briefly reached a median frequency of 6.41 Hz (Kolmogorov-Smirnov *Z* value: 1.9 ± 1.3), demonstrating that influx of extracellular Ca^2+^ is required for the effect of BDNF on mEPSC frequency.

Next, intracellular Ca^2+^ stores were depleted by bath application of the SERCA pump inhibitor thapsigargin (1 *μ*M) [34]. Under this condition, the magnitude of the BDNF effect on mEPSC frequency (0.87 ± 0.3 Hz versus 14.11 ± 3.5 Hz, *n* = 3) was significantly smaller than that observed under control conditions (*P* < 0.05). The median mEPSC frequency changed from 1.1 Hz to 3.7 Hz (Kolmogorov-Smirnov *Z* value was 6.6 ± 2.6) (Figure 3(b)). The temporal features of this significantly smaller response, which peaked at 102.5 ± 63.1 sec after BDNF application, and lasted 135 ± 90.37 sec, are not significantly different from control conditions. Such significantly smaller BDNF response in the presence of thapsigargin may reflect incomplete store depletion, or the contribution by capacitative Ca^2+^ entry triggered by store depletion.

Consistent with a presynaptic mechanism, the inclusion of 20 mM BAPTA in the neurons under recording did not affect the ability of BDNF to increase the mean mEPSC from 1.58 ± 0.59 Hz to 20.04 ± 4.73 Hz (*n* = 5, *P* = 0.0196) (Figure 3(c)). The median mEPSC frequency increased from 0.93 Hz to 8.68 Hz following BDNF application, and a Kolmogorov-Smirnov test yielded a *Z* value of 9.51 ± 1.93.

Taken together, these results suggest that an elevation of intracellular Ca^2+^ in presynaptic terminals mediated by both Ca^2+^ influx and mobilization of intracellular stores is necessary for BDNF to increase mEPSC frequency.

### 3.4. TRPC Channels Are Necessary for BDNF to Increase mEPSC Frequency

**Figure 4:**
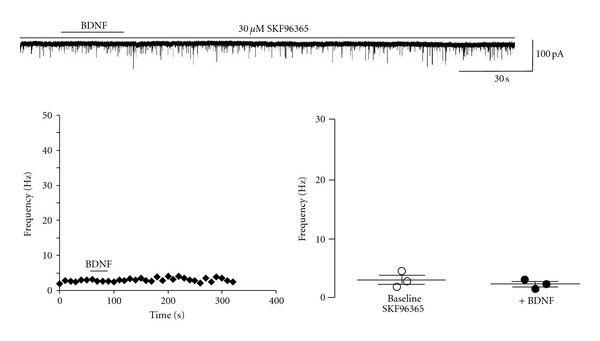
Inhibiting TRPC channels prevented the BDNF-induced increase in mEPSC frequency. Top: representative trace from a CA1 pyramidal cell in the presence of the SOC/TRPC channel inhibitor SKF96365 (30 *μ*M) prior to and following BDNF application; left: representative running average plot of mEPSC frequency in the presence of SKF96365; right: mean frequency of mEPSCs before and after BDNF in the continuous presence of SKF96365.

The requirement of both Ca^2+^ influx and intracellular Ca^2+^ stores suggested the involvement of TRPC channels, which are activated by the PLC cascade and mediated the activation of postsynaptic currents and dendritic Ca^2+^ signals by BDNF in CA1 and CA3 pyramidal neurons [10, 22, 35]. Bath application of the TRPC inhibitor SKF-96365 [36] completely prevented the effect of BDNF on mEPSC frequency (3.04 ± 2.29 Hz versus 2.29 ± 0.78 Hz; *n* = 3, *P* = 0.4256) (Figure 4(a)). The median mEPSC frequency changed from 2.75 Hz to 2.28 Hz after BDNF application (Kolmogorov-Smirnov test *Z* value 2.3 ± 1.9).

### 3.5. BDNF Rapidly Evokes FM Dye Destaining in the Presence of TTX

**Figure 5:**
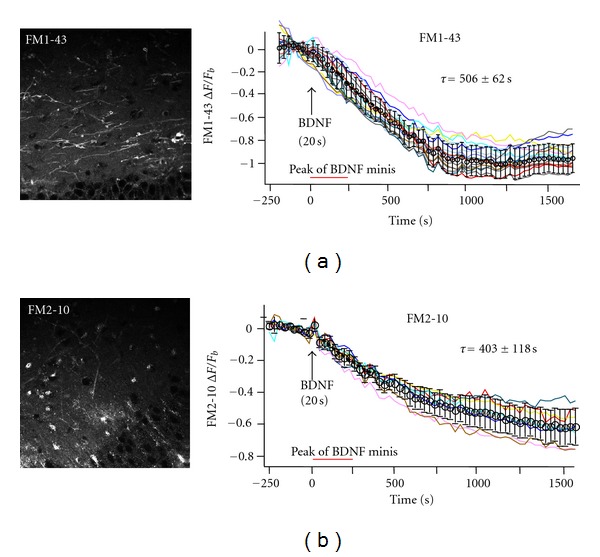
BDNF caused destaining of FM dyes in the presence of TTX. (a) Left: representative image of FM1-43 labeled puncta in hippocampal slice cultures by multiphoton excitation microscopy (840 nm excitation); right: BDNF caused destaining of FM1-43 labeled puncta with a *τ* = 506 ± 62 sec (8.4 min); *n* = 6 slices. (b) Left: representative image of FM2-10 labeled puncta in hippocampal slice cultures by multiphoton excitation microscopy (840 nm excitation), right: BDNF caused destaining of FM2-10 labeled puncta with a *τ* = 403 ± 118 sec (6.7 min); *n* = 4 slices.

To directly demonstrate that the elevated mEPSC frequency evoked by BDNF represents vesicular transmitter release, we performed multiphoton imaging of FM dyes in slice cultures. The total pool of recycling synaptic vesicles was labeled by exposing slices to a depolarizing solution (40 mM K^+^) containing 5 *μ*M FM1-43 (Figure 5(a)) [18, 26]. Following FM1-43 dye washout in a zero Ca^2+^ solution (to prevent FM1-43 destaining by spontaneous vesicle fusions), slices were imaged by multiphoton excitation microscopy (840 nm excitation) in Ca^2+^-containing aCSF with TTX to block Na^+^-dependent action potentials. BDNF was applied to apical dendrites in area CA1 using a Picospritzer in the same manner as for whole-cell recordings of mEPSCs, and FM1-43 fluorescence intensity was followed over time (0.9 frames per second, 30 min movies). Figure 5(a) shows a representative example of BDNF-induced FM1-43 destaining in the presence of TTX to block spike-dependent transmitter release. The rate of the exponential decay of FM1-43 fluorescence intensity immediately following BDNF application was 506 ± 62 sec (*n* = 6 slices). These observations were confirmed with FM2-10, a styryl lipophilic dye with lower membrane-binding affinity (Figure 5(b)). The exponential decay rate of FM2-10 intensity evoked by BDNF in TTX was 403 ± 118 sec (*n* = 4 slices), which was not significantly different that that observed using FM1-43 (*P* > 0.05). These results show that BDNF induces FM dye loss by a vesicular fusion mechanism that is independent of the membrane affinity and molecular size of FM1-43 and FM2-10 dyes. Furthermore, these imaging results directly demonstrate that the elevated mEPSC frequency caused by BDNF representS vesicular transmitter release from presynaptic terminals.

## 4. Discussion

The present study demonstrates that the BDNF-induced increase in mEPSC frequency recorded in hippocampal CA1 pyramidal neurons represents Ca^2+^-dependent vesicle release from presynaptic terminals. The BDNF-induced increase in mEPSC frequency is prevented by bath application of the tyrosine kinase inhibitor k-252a. Depletion of intracellular Ca^2+^ stores with thapsigargin also inhibited the BDNF-induced mEPSC frequency increase, as did the removal of extracellular Ca^2+^. These results demonstrate the critical requirement of TrkB-initiated Ca^2+^-dependent signaling in the BDNF-induced increase in mEPSC frequency.

One of the signaling cascades activated by Trk receptors, the hydrolysis of PIP_2_ by PLC*γ* leading to IP3 formation, has long been known to cause intracellular Ca^2+^ mobilization in neurons [37]. Indeed, BDNF increased Ca^2+^ levels within presynaptic terminals of *Xenopus* neuromuscular junctions in culture, leading to a transient enhancement of transmitter release that was dependent on extracellular Ca^2+^ levels and presynaptic depolarization [38, 39]. Similarly, extracellular Ca^2+^ was required for BDNF to increase the frequency of mEPSCs and somatic Ca^2+^ levels in cultured hippocampal neurons [40]. In addition to well-established voltage- and ligand-gated mechanisms of Ca^2+^ influx, Ca^2+^ signals mediated by depletion of intracellular Ca^2+^ stores are fundamental component of neuronal Ca^2+^ signaling. In non-excitable cells, the depletion of Ca^2+^ stores activates plasma membrane channels often called store-operated channels (SOCs), allowing Ca^2+^ entry for the replenishment of emptied stores; this process is called capacitative Ca^2+^ entry [41, 42]. Recent evidence suggests that cultured hippocampal neurons exhibit such capacitative Ca^2+^ entry [23, 43, 44]. Furthermore, we recently demonstrated that BDNF evokes postsynaptic currents and dendritic Ca^2+^ signals in CA1 and CA3 pyramidal neurons through the activation of TrkB/PLC and mediated by TRPC3-containing channels [10, 22, 35].

Here, we present evidence consistent with the model that the source of Ca^2+^ that contributes to the BDNF-induced increase in mEPSC frequency originates from presynaptic ER stores. The contribution of intracellular ER stores as a source of Ca^2+^ to asynchronous (spontaneous) quantal transmitter release has been a highly debated issue. Although some reports demonstrate that store depletion in hippocampal pyramidal neurons leads to Ca^2+^-induced Ca^2+^ release (CICR) and subsequent paired-pulse facilitation of excitatory postsynaptic potentials [23], other reports are to the contrary. Using thapsigargin, which empties ER stores by poisoning the SERCA pump, or ryanodine, which blocks ryanodine receptors, it has been demonstrated that ER stores and CICR do not affect paired-pulse facilitation of excitatory postsynaptic potentials [45]. These studies were conducted at four different excitatory synapses in the brain: hippocampal CA3-CA1 synapses; mossy fiber synapses between the dentate granule cells and CA3 pyramidal cells; synapses between CA3 neurons; cerebellar parallel fiber to Purkinje cell synapses [45]. Still others have shown that immediate mEPSC frequency increases are dependent upon mitochondrial excitatory postsynaptic potentials [46].

Our studies have directly demonstrated that the elevated frequency of mEPSCs evoked by brief and local BDNF application indeed reflects vesicular fusion events from presynaptic terminals. We also attempted to determine whether the vesicular fusions underlying FM1-43 loss from terminals represented kiss-and-run or full-fusion events by comparing them to FM2-10 loss, which has a slower departition rate from lipid membranes [47, 48]. However, the kinetics of BDNF-induced FM2-10 destaining was not different than that of FM1-43. This suggests that BDNF caused dye loss either via full-fusion events or through fusion pores with a permeability for styryl dyes that is not affected by the molecular sizes of FM1-43 and FM2-10. Recently, it has been determined that the FM family of dyes is not suitable for determining whether vesicle release occurs via full-fusion or kiss-and-run events [49].

Taken together, these results demonstrate that BDNF increases mEPSC frequency by enhancing vesicular fusion through a presynaptic Ca^2+^-dependent mechanism involving TrkB receptors, Ca^2+^ stores, and TRPC channels.
